# Dose–response effects of a mixed condensed and hydrolyzable tannin extract on methane production and diet digestibility using the in vitro gas production technique

**DOI:** 10.1093/jas/skaf389

**Published:** 2025-11-04

**Authors:** Jordan M Adams, Clarice M Francis, Mingyung Lee, Marcia H M R Fernandes, Luis O Tedeschi

**Affiliations:** Department of Animal Science, Texas A&M University, College Station, TX, 77843-2471; Department of Animal Science, Texas A&M University, College Station, TX, 77843-2471; Department of Animal Science, Texas A&M University, College Station, TX, 77843-2471; Department of Animal Science, Texas A&M University, College Station, TX, 77843-2471; Department of Animal Science, Texas A&M University, College Station, TX, 77843-2471

**Keywords:** beef cattle, fermentation, in vitro, methane, tannin extract

## Abstract

Several studies have evaluated the impact of isolated condensed or hydrolyzable tannin extract (TE) supplementation for beef cattle on methane (CH_4_) mitigation and metabolic functions, but fewer have evaluated their combination. Our objective was to investigate changes in in vitro fermentation dynamics, CH_4_ production, neutral detergent fiber digestibility (ivNDFD), and ruminal volatile fatty acid (VFA) concentrations in response to the inclusion rate of a TE blend (Silvafeed ByPro; SILVATEAM). Inclusion of TE at 0.0%, 0.3%, 0.6%, and 0.9% DM (TE_0.0_, TE_0.3_, TE_0.6_, and TE_0.9_), within a total mixed ration, was evaluated using four in vitro incubations. Fresh rumen inoculum from two ruminally cannulated donor steers was used for each incubation. Mixed-effects polynomial regression models, with incubations as the random effect, were used for dose–response analysis. The inclusion rate did not affect total gas production (*P *≥ 0.67) determined from an exponential model. The fractional rate of exponential gas production decreased linearly (*P *< 0.01) with increasing inclusion. The lag time for initiating gas production displayed quadratic (*P *< 0.01) and cubic (*P *= 0.01) patterns. Based on the log 2-pool model, cumulative gas production from both carbohydrate pools was influenced by inclusion rate in a cubic (*P *≤ 0.01) fashion, but in opposite directions. Cumulative gas production for the nonfiber carbohydrate pool peaked at 0.05% DM inclusion and reached a minimum at TE_0.9_, while that of the fiber carbohydrate (FC) pool was minimized at 0.21% DM and peaked at TE_0.9_. There was a quadratic (*P *= 0.04) effect for the lag time to begin fermentation of the FC pool. Total CH_4_ production displayed a cubic (*P *= 0.04) pattern in response to TE inclusion, with minimum production at 0.18% of DM. Production of CH_4_ per g of fermentable organic matter tended (*P *= 0.06) to follow the same cubic pattern. There was no influence of inclusion rate on ivNDFD (*P *≥ 0.68) or computed energy values (*P *≥ 0.67). Ruminal pH and total VFA concentration were not affected by treatment (*P ≥ *0.11), but propionate linearly decreased (*P *= 0.02) and the acetate: propionate ratio linearly increased (*P *< 0.01) with increasing TE inclusion. Our results suggest that inclusion of TE at 0.18% of DM has the greatest potential for in vitro CH_4_ mitigation. Further research is warranted to determine the dose–response relationships between the supplementation rate of this TE and in vivo CH_4_ production and ruminal parameters.

## Teaser

A low inclusion rate of a mixed condensed and hydrolyzable tannin extract may have the greatest potential to reduce methane production from beef cattle consuming a total mixed ration.

## Introduction

Meat production is a major source of high-quality protein in human diets (Mottet et al., 2017). However, as the global population continues to grow, there is increasing pressure to improve the efficiency and sustainability of meat production, particularly in the beef sector (Tedeschi et al., 2015; Flachowsky et al., 2017). Beef cattle can efficiently convert low-quality feedstuffs into high-quality products for human consumption; however, the gaseous byproducts (e.g., methane) naturally produced during fermentative processes have been scrutinized for their potential environmental impacts related to global warming (Tedeschi and Beauchemin, 2023). Methane (CH_4_) production has been a primary focus for reducing greenhouse gas (GHG) emissions because it has a 100-year global warming potential that is 27 times greater than that of carbon dioxide (Forster et al., 2021). Additionally, the CH_4_ produced from enteric fermentation accounted for 32.5% of the total GHG emissions from the agricultural sector in the United States in 2022 (EPA, 2024). Although that only equates to approximately 3% of the country’s total emissions (Tedeschi and Beauchemin, 2023), there remains a push to reduce CH_4_ production from ruminant animals. Nutritional management strategies, such as the use of feed additives, have been widely studied as a viable option for mitigating GHG emissions in the livestock industry. However, feed additives developed to decrease CH_4_ emissions from beef cattle must selectively reduce CH_4_ production while avoiding reductions in total gas production or fermentation, which could negatively impact animal performance (Naumann et al., 2017).

Tannins are naturally occurring plant defense compounds that act against predators and pathogens (Tedeschi et al., 2021; Poli et al., 2025). Due to their inherent ability to modulate rumen fermentation, tannins have also been studied as a dietary supplement in beef cattle production. In ruminant animals, tannins can form complexes with proteins and carbohydrates, which may improve nitrogen utilization and reduce CH_4_ production (Patra and Saxena, 2011). Legumes or other plants containing tannins may be directly fed to the animal, but utilizing the tannin extract (TE) from the plant at a known concentration may be more beneficial to yield the desired benefits. Condensed tannins (CT) and hydrolyzable tannins (HT) are two primary types of tannins that are naturally occurring in plants and have been studied for use in beef cattle production (Poli et al., 2025). Although several studies have evaluated the effects of isolated CT or HT as a feed additive, supplementing a combination of both tannin types may yield synergistic effects. Jayanegara et al. (2015) compared the effects of purified individual HT and CT on in vitro CH_4_ production and ruminal fermentation and reported a 6.6% and 3.5% reduction in CH_4_ production for HT (chestnut; *Castanea* sp.) and CT (quebracho; *Schinopsis balansae*), respectively, at a dose of 1.0 mg/mL compared to the control. There are concerns of potential toxicity when supplementing HT alone, so supplementing beef cattle with a combination of HT and CT may reduce the toxicity risk, due to a lower HT dose required, while also having greater CH_4_ mitigation effects (Jayanegara et al., 2015). Chen et al. (2021) observed a greater reduction in in vitro CH_4_ production when alfalfa silage was treated with a combination of HT and CT at both low (1% of DM each) and high (2.5% of DM each) doses compared to the individual tannins. When dairy heifers were supplemented with increasing doses of TE (mixed CT and HT) at up to 0.3% of dry matter intake (DMI), Schilling-Hazlett et al. (2024) observed no effect on CH_4_ emissions, raising the hypothesis that a dose of the TE containing both CT and HT higher than 0.3% of DMI would be necessary to reduce CH_4_ emissions. In addition, Aguerre et al. (2016) evaluated the supplementation of a mixed TE at up to 1.8% of DM on ruminal fermentation and observed the greatest benefits at the 0.45% of DM dose. Ultimately, the dose–response relationship and the optimal dose of a TE containing both CT and HT for reducing CH_4_ emissions from cattle remains unclear. We hypothesized that there would be a dose–response relationship between TE inclusion and in vitro CH_4_ production. Our objective was to investigate the changes in CH_4_ production and feed digestibility in response to varying inclusion rates of a TE (blended CT and HT), using the in vitro gas production (IVGP) technique.

## Materials and Methods

All animals and procedures used in this experiment were reviewed and approved by the Institutional Animal Care and Use Committee (AUP #2024-0111) at Texas A&M University.

### Treatments and data collection

We evaluated the effects of a blended quebracho condensed (*Schinopsis lorentzii*) and hydrolyzable (tannic acid) TE (Silvafeed ByPro; SILVATEAM, San Michele Mondovi, Italy) at 0.0%, 0.3%, 0.6%, and 0.9% of dietary dry matter (DM; TE_0.0_, TE_0.3_, TE_0.6_, and TE_0.9_) on in vitro fermentation dynamics in a randomized complete block design, where percentages refer to the total TE. The TE contained 40.17% total CT (TCT), composed of 19.08% extractable, 20.62% protein-bound, and 0.46% fiber-bound CT (Terrill et al., 1992; Wolfe et al., 2008). In addition, the bioreactivity was determined as protein precipitable phenolics (PPP; Hagerman and Butler, 1978; Naumann et al., 2014), and the TE contained 24.75% PPP. Although the inclusion rates in this study were based on the percentage of the total TE, it is difficult to make interstudy comparisons as the percentage of total TE is not related to the CT and HT content or the bioreactivity of the product (Muir et al., 2023). Therefore, we have converted the inclusion rates to both TCT and PPP basis to aid in interstudy comparisons. The inclusion rates correspond to 0.0 (TE_0.0_), 0.11 (TE_0.3_), 0.21 (TE_0.6_), and 0.32% (TE_0.9_) of TCT and 0.0 (TE_0.0_), 0.06 (TE_0.3_), 0.13 (TE_0.6_), and 0.19% (TE_0.9_) on a PPP basis, which is related to the bioreactivity of the TE.

The in vitro gas production (IVGP) technique, as described by Tedeschi et al. (2009), with modifications discussed by Tedeschi and Fox (2020), was used in this study. Four in vitro incubations were performed within a fermentation chamber maintained at 39 °C, equipped with a multiplate stirrer, and the capacity to house 32 160-mL Wheaton bottles. Within each incubation, each treatment was randomly assigned to seven bottles, and four bottles were used as blanks (without the addition of substrate). The fermentative substrate utilized was a total mixed ration (TMR), with fresh samples being air dried at 55 °C for 48 h, or until weight loss ceased, before being ground to pass through a 2-mm sieve. A 500-g DM subsample of the ground feed was separated into individual storage containers for each inclusion rate. For each of the TE treatments, the appropriate quantity of TE was premixed into the ground feed using an electric rotator (Dry Powder Rotator; Glas-Col LLC, Terre Haute, IN) for 20 min. A subsample of the basal TMR was sent to Cumberland Valley Analytical Services (Waynesboro, PA) for chemical analysis (Table 1). Two ruminally cannulated British-crossbred steers (792 ± 17.0 kg; approximately 8 years of age) consuming ad libitum bermudagrass hay were used as rumen fluid donors. Approximately 500 mL of rumen inoculum was collected from each animal via ruminal cannula into a 600 mL thermos and transported back to the laboratory. Immediately following collection, pH and redox potential of the inoculum were recorded (6.34 and –351.8 mV, respectively, on average).

**Table 1.** Ingredient and chemical composition of the total mixed ration utilized as the fermentation substrate

Items^1^	Basal diet, %
**Ingredient composition, %DM**	
** Bermudagrass hay**	35.00
** Cracked corn**	39.50
** Dried distillers’ grain**	16.00
** Molasses**	6.00
** Mineral**	3.50
**Chemical composition^2^**	
** DM, %**	87.38
** CP, % DM**	11.30
** Soluble protein, % CP**	25.32
** aNDF, % DM**	35.88
** ADF, % DM**	16.42
** Lignin, % DM**	2.81
** Crude fat, % DM**	3.71
** Sugar, % DM**	8.62
** Starch, % DM**	29.25
** Ash, % DM**	6.28
** Calcium, % DM**	0.73
** Phosphorus, % DM**	0.36
** TDN, % DM**	72.97
** NE_m_, Mcal/kg**	1.66
** NE_g_, Mcal/kg**	1.05
** ME, Mcal/kg**	2.56

1 Items are feed ingredients, and the chemical composition of the basal diet was evaluated by Cumberland Valley Analytical Services (Waynesboro, PA).

2 DM: dry matter; CP: crude protein; aNDF: neutral detergent fiber with amylase and sodium sulfite; ADF: acid detergent fiber; TDN: total digestible nutrients; NE_m_: net energy for maintenance; NE_g_: net energy for gain; ME: metabolizable energy.

The preparation procedure for in vitro incubations has been described in depth by Crossland et al. (2018) and Dias Batista et al. (2020). Briefly, Wheaton bottles were prepared with equal-sized magnetic stir bars and 200 mg of dried and ground substrate containing the treatments. Additionally, 2 mL of distilled H_2_O was added to dampen the sample and prevent particle scattering, and 14 mL of Goering and Van Soest (1970) buffering media was added to each bottle, under continual flushing of CO_2_ to maintain an oxygen-reduced environment. The bottles were then sealed using butyl rubber stoppers coated with petroleum jelly and crimp seals before being transferred to the incubation chamber to reach rumen temperature. Fresh rumen inoculum was filtered through glass wool to remove any feed particles and then transferred into a Wheaton bottle flushed with CO_2_. Then, 4 mL of rumen inoculum was injected into each bottle via syringe and needle. The internal pressure was equilibrated for all bottles following inoculation by inserting a needle into the rubber stoppers for approximately 5 s before the pressure sensors were inserted and data recording was initiated. Upon initiation of data recording, the pressure inside each bottle was recorded at 5-min intervals for 48 h using PicoLog software (release 5.22.1; Pico Technology, Tyler, TX), with fermentation profiles being plotted in real-time.

Kinetic analyses of the 48-h fermentation were plotted and evaluated using nonlinear functions, in which the lowest sum of square errors (Schofield et al., 1994) was used to select the functions using the GasFit (version 3.10.9243.20681; https://www.nutritionmodels.com/gasfit.html) system. GasFit utilizes specific R scripts for the convergence of gas production data using *nls* and *port* algorithms (Fox et al., 1978; Gay, 1990; Chambers and Bates, 1992). The data output from the Gasfit system included total gas production (mL), fractional rate of fermentation (1/h), and lag time (h) from exponential curves, as well as asymptote cumulative gas production of the nonfiber carbohydrate (NFC) pool (mL), fractional rate of fermentation of the NFC pool (1/h), lag time (h), asymptote cumulative gas production of the fiber carbohydrate (FC) pool (mL), and fractional rate of fermentation of the FC pool (1/h) from the logarithmic two-pool nonlinear function (Tedeschi and Fox, 2020). In addition, total digestible nutrients (TDN) and metabolizable energy (ME) values were calculated using empirical equations (Tedeschi et al., 2009; NASEM, 2016).

### Sample analyses

Following the 48-h incubation, bottles were immediately placed on ice to cease fermentation. A subsample of headspace gas (10 mL) was removed from each bottle and analyzed for methane (CH_4_) concentration via gas chromatography (GOW-MAC Series 580; Gow-Mac Instrument, Bethlehem, PA) according to Allison et al. (1992), with argon as the carrier gas and a sample size of 0.5 mL. The post-fermentation pH and redox potential of the fermentation broth were recorded for each bottle using a benchtop meter (Thermo Scientific Orion Star A210; Thermo Fisher Scientific, Waltham, MA). Additionally, the replicate bottles for each treatment were randomly preassigned to one of two final analyses. Four of the seven replicates from each treatment and two blanks had 40 mL of neutral detergent solution (ANKOM Technology, Macedon, NY; Van Soest et al., 1991) added to each bottle before being resealed and autoclaved for 15 min at 121 °C. The contents of those bottles were then filtered using Whatman 54 filter paper to retain the feed residue and dried at 105 °C for 24 h to calculate in vitro neutral detergent fiber digestibility (ivNDFD; Pell and Schofield, 1993). Two 1.5 mL subsamples of the fermentation broth from the remaining three replicates from each treatment and two blanks were collected and stored at −20°C for volatile fatty acid (VFA) analysis. Concentrations of VFA were determined according to Weimer et al. (1991) using high-performance liquid chromatography (Shimadzu Scientific Instruments, Columbia, MD) equipped with a temperature-controlled autosampler (Nexera SIL-30AC UHPLC Cooled Autosampler, Shimadzu Scientific Instruments, Columbia, MD), a forced-air column oven (CTO-20A, Shimadzu Scientific Instruments, Columbia, MD), and a UV absorbance detector (SPD-20A UV Detector; Shimadzu Scientific Instruments, Columbia, MD). An Aminex HPX-87H column (Bio-Rad HPX-87H, 300 mm × 7.8 mm i.d.; Bio-Rad Laboratories Inc., Hercules, CA) and its guard column (Bio-Rad Cation H, Bio-Rad Laboratories Inc., Hercules, CA) were used to separate peaks at 210 nm absorbance over 100 min retention time.

### Statistical analyses

All statistical analyses were performed using R 4.4.1 (R Core Team, Vienna, Austria). Data for all variables were tested for outliers by evaluating the internally calculated studentized residuals, and data points with a studentized residual outside the range of ±3 absolute values were removed (Kutner et al., 2005). A total of nine data points were removed, with each treatment having at least one outlier. Dose–response relationships were evaluated using mixed-effects polynomial regression models fitted with the *lmer* function (lme4 package) to account for the random intercept of incubation (Bates et al., 2015). Each incubation bottle was considered as the experimental unit for all evaluated variables. Raw powers of inclusion rate were used in the polynomial terms to reflect actual dose spacing. Estimated marginal means were calculated to interpret the effects of inclusion rates. Akaike’s Information Criterion (AIC) was used to select the polynomial model providing the best fit to the data, with the lowest value considered the most adequate. Polynomial terms were considered significant at *P *≤ 0.05, and tendencies were assumed at 0.10 ≥ *P *> 0.05. Inclusion rates corresponding to the minimum or maximum predicted response within the observed treatment range were identified by the critical points of the fitted polynomial models.

## Results

The inclusion rate of TE did not influence total gas production (*P *≥ 0.67; Table 2). There was a dose–response pattern (linear *P *< 0.01, quadratic *P *< 0.01, cubic *P *< 0.01) for the fractional rate of exponential gas production, but the linear model best fit the data, with the fractional rate decreasing as TE inclusion rate increased. Additionally, the lag time for initiating gas production was influenced by TE inclusion rate in a quadratic (*P* < 0.01) and cubic (*P *= 0.01) fashion, with lag time reaching a maximum at TE_0.3_ and then decreasing to a minimum at TE_0.9_.

**Table 2.** Dose–response relationship of tannin extract inclusion upon in vitro gas production dynamics

	Treatment^1^					*P*-values^2^		
Item^3^	TE_0.0_	TE_0.3_	TE_0.6_	TE_0.9_	SEM^4^	L	Q	C
**Exponential**								
** TGP, mL**	29.77	29.76	29.45	29.63	0.569	0.67	0.89	0.93
** kd, %/h**	10.24	9.99	9.63	9.22	0.690	<0.01	<0.01	<0.01
** Lag time, h**	0.36	0.50	0.42	0.28	0.138	0.21	<0.01	0.01
**Log 2-pool**								
** Asymptote (P1), mL**	20.29	20.09	19.53	19.08	1.060	<0.01	<0.01	0.01
** kd P1, %/h**	10.68	10.57	10.59	10.22	1.236	0.23	0.42	0.57
** Lag time to P2, h**	1.19	1.41	1.39	1.24	0.100	0.55	0.04	0.10
** Asymptote (P2), mL**	10.22	10.11	10.43	10.97	0.785	<0.01	<0.01	<0.01
** kd P2, %/h**	2.78	2.78	2.70	2.73	0.267	0.30	0.56	0.59
** Exp. kd equivalent, %/h**	4.73	4.73	4.58	4.64	0.545	0.32	0.57	0.61
**Methane, mg**	2.86	2.73	3.04	2.83	0.102	0.59	0.70	0.04
**Methane, mM**	0.18	0.17	0.19	0.18	0.006	0.59	0.70	0.04
**Methane, mg/g FOM**	15.01	14.26	15.78	14.75	0.548	0.70	0.81	0.06
**Methane, mg/g NFC**	32.76	31.12	34.44	32.19	1.196	0.70	0.81	0.06
**Methane, mg/g NDF**	39.10	37.14	41.12	38.43	1.427	0.70	0.81	0.06
**Methane, % of total gas**	16.00	15.34	17.45	16.18	0.730	0.26	0.40	0.02
**ivNDFD, % (48 h)**	72.45	75.48	74.49	71.70	5.172	0.87	0.68	0.85
**TDN, %**	72.76	72.87	72.66	72.81	0.509	0.93	0.97	0.67
**ME, Mcal/kg (TDN, 4%)**	2.63	2.63	2.62	2.63	0.018	0.93	0.97	0.67

1 Treatment = levels of tannin extract incubated during in vitro fermentation; TE_0.0_, 0.0% of DM; TE_0.3_, 0.3% of DM; TE_0.6_, 0.6% of DM; TE_0.9_, 0.9% of DM.

2 L: linear; Q: quadratic; C: cubic.

3 TGP, total gas production of the exponential nonlinear function; kd, fractional rate of gas production of the exponential nonlinear function; lag time, time required to begin fermentation; asymptote (P1), accumulative gas production of nonfiber carbohydrate (NFC) pool; kd (P1), fractional rate of gas production of NFC pool; lag time to P2, time required to begin fermentation of fiber carbohydrate (FC) pool; asymptote (P2), accumulative gas production of FC pool; kd P2, fractional rate of gas production of FC pool; Exp. kd equivalent, exponential decay rate of digestion; FOM, fermentable organic matter; ivNDFD, in vitro neutral detergent fiber digestibility.

4 Group variances estimated separately, the largest SEM is reported.

**Figure 1.**
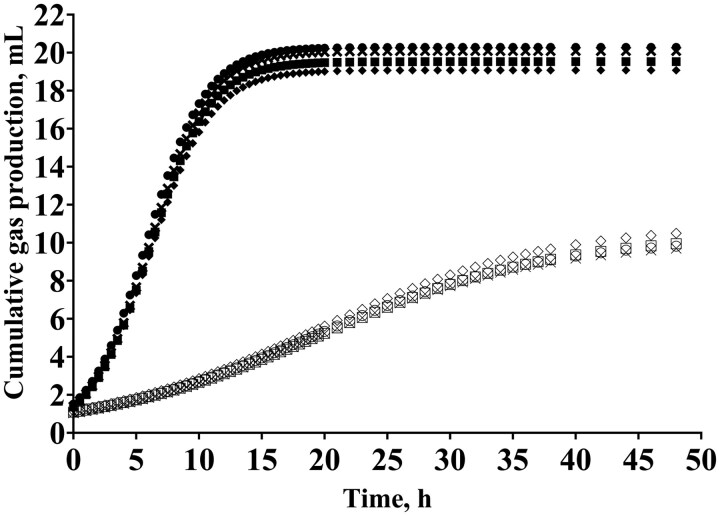
Effect of tannin extract (TE) inclusion rate on the log 2-pool cumulative gas production of the nonfiber carbohydrate (NFC; filled shapes) and fiber carbohydrate (FC; unfilled shapes) pools. Circle = TE at 0.0% of DM, X = TE at 0.3% of DM, square = TE at 0.6% of DM, diamond = TE at 0.9% of DM.

**Figure 2.**
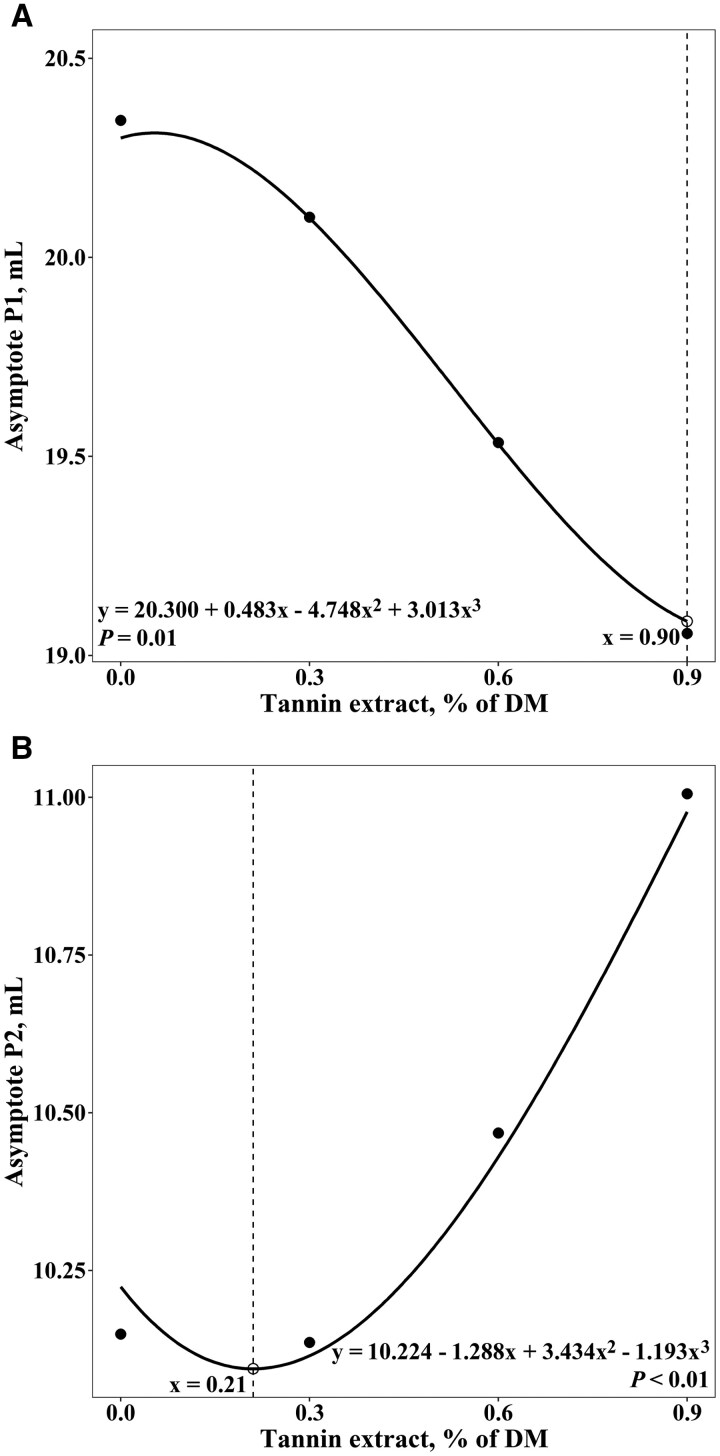
Dose–response relationship of tannin extract inclusion on (A) cumulative gas production of the nonfiber carbohydrate pool (Asymptote P1) and (B) cumulative gas production of the fiber carbohydrate pool (Asymptote P2).

**Figure 3.**
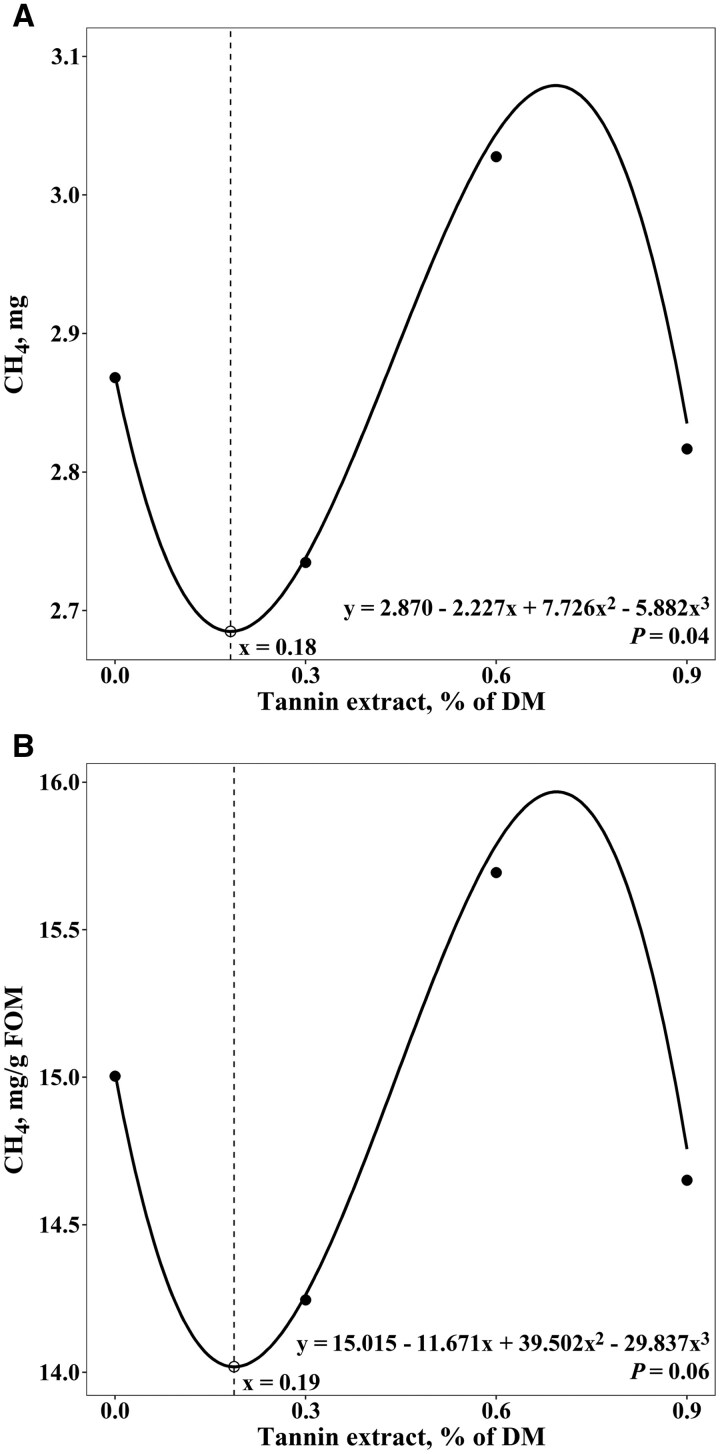
Dose–response relationship of tannin extract inclusion on (A) total methane (CH_4_) production and (B) CH_4_ production per gram of fermentable organic matter (FOM).

Figure 1 displays the overall pattern of cumulative gas production from the logarithmic 2-pool function based on treatment means. There was a dose–response pattern (linear *P *< 0.01, quadratic *P *< 0.01, cubic *P *= 0.01) for the cumulative gas production of the NFC pool (Table 2). The cubic model best fit the data, indicating a peak in cumulative gas production of the NFC at 0.05% of dietary DM inclusion and a minimum at TE_0.9_ (Figure 2A). The cumulative gas production of the FC pool also displayed dose–response patterns (linear *P *< 0.01, quadratic *P *< 0.01, cubic *P *< 0.01) in response to TE inclusion rates (Table 2), but in the opposite direction. In this case, the cubic model indicated that cumulative gas production of the FC pool was minimized at an inclusion rate of 0.21% of DM and peaked at TE_0.9_ (Figure 2B). There was a quadratic effect (*P *= 0.04) for the lag time required to begin fermentation of the FC pool (Table 2), with the lag time at a minimum at TE_0.0_, increasing up to 0.47% of dietary DM, and decreasing thereafter. However, there was no influence of TE inclusion rate on the fractional rates of gas production of the NFC or FC pools (*P *≥ 0.23), or the exponential decay rate of digestion (*P *≥ 0.32). Total CH_4_ production displayed a cubic (*P *= 0.04) pattern in response to TE inclusion rate. More specifically, the cubic model indicated the minimum CH_4_ production at an inclusion rate of 0.18% of DM and the maximum at 0.69% of DM (Figure 3A). In addition, CH_4_ produced per gram of fermentable organic matter (FOM) tended (*P *= 0.06) to follow the same cubic pattern (Figure 3B), as well as CH_4_ produced per gram of NFC and NDF (*P *= 0.06). When expressed as a percentage of total gas production, there was also a cubic (*P *= 0.02) effect on CH_4_ production. However, the TE inclusion rate did not influence ivNDFD (*P *≥ 0.68) or computed TDN and ME (*P *≥ 0.67) values.

Ruminal pH was not impacted by the TE inclusion rate (*P *≥ 0.21), but redox potential displayed linear (*P *= 0.01) and quadratic (*P *= 0.04) patterns (Table 3), although the quadratic model best fit the data with the lowest redox potential at TE_0.9_. The ruminal concentration of propionate decreased linearly (*P *= 0.02) with increasing TE inclusion, and a tendency (*P *= 0.06) to display a quadratic pattern as well. Additionally, there was a dose–response relationship (linear *P *< 0.01, quadratic *P *< 0.01, cubic *P *< 0.01) between TE inclusion and the acetate-to-propionate ratio. However, the pattern was primarily linear, based on the model fit. There were no differences in the concentrations of other VFA (*P *≥ 0.19) or the total concentration of VFA (*P *≥ 0.11).

**Table 3.** Dose–response relationship of tannin extract inclusion upon ruminal parameters

	Treatment^1^					*P*-values^2^		
Item	TE_0.0_	TE_0.3_	TE_0.6_	TE_0.9_	SEM^3^	L	Q	C
**pH**	6.18	6.17	6.18	6.19	0.039	0.21	0.25	0.33
**Redox, mV**	-320.42	-324.62	-324.84	-327.09	5.254	0.01	0.04	0.07
**Total VFA, mmol/L**	94.10	94.31	89.43	85.45	4.992	0.11	0.25	0.42
** Acetate, mmol/L**	51.14	51.65	48.88	47.44	3.232	0.20	0.42	0.59
** Propionate, mmol/L**	24.95	24.42	22.90	21.04	2.023	0.02	0.06	0.14
** Butyrate, mmol/L**	10.14	10.31	9.95	9.78	0.682	0.52	0.77	0.89
** Isobutyrate, mmol/L**	2.20	1.95	1.88	1.94	0.147	0.19	0.24	0.42
** Valerate, mmol/L**	3.31	3.78	3.72	3.24	0.415	0.86	0.21	0.39
** Isovalerate, mmol/L**	2.71	2.75	2.57	2.47	0.263	0.19	0.40	0.57
**Acetate: propionate**	2.10	2.17	2.17	2.26	0.206	<0.01	<0.01	<0.01
**Lactic, mmol/L**	0.14	0.09	0.11	0.22	0.046	0.19	0.05	0.12

1 Treatment = levels of tannin extract incubated during in vitro fermentation; TE_0.0_, 0.0% of DM; TE_0.3_, 0.3% of DM; TE_0.6_, 0.6% of DM; TE_0.9_, 0.9% of DM.

2 L: linear; Q: quadratic; C: cubic.

3 Group variances are estimated separately, and the largest SEM is reported.

## Discussion

A reduction in CH_4_ production without impacting total gas production is important to prevent a subsequent reduction in animal performance (Naumann et al., 2017). Although there were indications of total CH_4_ production decreasing to a minimum at 0.18% of DM inclusion, total gas production remained similar among all rates. When Chen et al. (2021) incubated alfalfa silage that was pretreated with varying levels and combinations of chestnut and quebracho TE, they observed a reduction in both in vitro gas and CH_4_ produced per gram of DM, regardless of tannin type and level of inclusion. However, their study evaluated 2% of DM of chestnut TE or a combination of chestnut and quebracho TE at 1% of DM each as their lowest doses, which are higher than those evaluated in the current study. Aside from the tannin types and doses, the contrasting results could be related to the different fermentative substrates used among the studies, with their study incubating alfalfa silage and ours incubating a TMR. Alfalfa contains saponins, which are phytochemicals that may also cause a reduction in CH_4_ production (Tedeschi et al., 2021), so the results of Chen et al. (2021) may not be solely attributable to the TE inclusion levels. Schilling-Hazlett et al. (2024) did not observe a significant difference in daily CH_4_ production when dairy heifers were supplemented with the same commercial TE product as the current study at a rate of up to 0.3% of DMI, but the 0.3% supplementation rate showed the greatest potential for reducing CH_4_ production. Two possible mechanisms were proposed by Tavendale et al. (2005) for CH_4_ reduction by CT extract supplementation: (1) indirect inhibition via a decrease in the ruminal H_2_ pool due to reduced fiber degradation, and (2) direct inhibition of methanogen growth. When comparing the effects of purified individual CT and HT on CH_4_ production, Jayanegara et al. (2015) concluded that CT might reduce CH_4_ production primarily at the expense of fiber digestibility, whereas HT act mainly through direct inhibition of methanogens. In the current study, the decrease in CH_4_ production observed at 0.18% of dietary DM was not associated with a reduction in ivNDFD. Although this suggests that direct inhibition of methanogens may have been the primary mechanism of action, microbial growth was not measured in the present study. The cubic pattern indicating an increase in CH_4_ production at the 0.6% of DM level was unexpected and challenging to explain for analyses conducted using in vitro systems. Getachew et al. (2008) observed a reduction in the intrinsic rate of gas production and an increase in asymptotic gas production when tannic and gallic acids were incubated with alfalfa hay at rates up to 60 g/kg DM. Although CH_4_ production was not measured in their study, their findings are similar to those in our study and may indicate degradation or toleration of HT by ruminal microbes and a subsequent shift in fermentation pathways (Murdiati et al., 1992; Getachew et al., 2008;). There may be several factors causing the reduction in CH_4_ production, acting at different times to result in the overall curvature pattern. For instance, the patterns of decreasing cumulative gas production from the NFC pool and increasing cumulative gas production from the FC pool, together with the decreased lag time to begin FC pool gas production, suggest that fermentation of the NFC fraction may have been limited initially, while that of the FC pool was accelerated. Therefore, the CH_4_ production at increasing inclusion levels may have been from fermentation of the FC pool initially, which aligns with the pattern of decreasing propionate concentration. Nevertheless, the inclusion of TE influenced CH_4_ yield relative to the fermentable NFC and NDF in a similar manner, with minimization at a dose of 0.19% of DM. Ultimately, there are several factors, beyond concentration, that may influence the ability of TE to reduce CH_4_ production, including molecular structure and weight, stereochemistry, hydroxylation, and functional groups (Naumann et al., 2013). Thus, TE concentration alone may not be a reliable predictor of its bioactivity (Naumann et al., 2013; Tedeschi and Fox, 2020). In addition, the analysis of microbial communities in the ruminal fluid would be beneficial for a comprehensive understanding of the mechanism of action of TE on CH_4_ production and the dose-dependent response. In theory, a reduction in CH_4_ production should allow for greater ME to be available for the animal because less gaseous energy is lost during fermentation. The computed TDN and ME were 72.77% and 2.63 Mcal/kg on average, respectively. In our case, the potential benefit of CH_4_ energy loss may have been offset by a numerical decrease in total VFA concentration (from 94.3 to 85.5 mmol/L).

The fractional rate of gas production of the exponential function decreased in a linear fashion, from 10.24% to 9.22% per hour, with increased TE inclusion. In agreement with our findings, Getachew et al. (2008) reported a linear reduction in the intrinsic rate of gas production when alfalfa hay was incubated in vitro with tannic acid at 2%–6% of DM and with quebracho TE at 6%–15% of DM. Similarly, a reduction in the rate of gas production was observed when a corn silage-based diet was incubated in vitro with the same TE used in the current study at 0.15% of DM (He et al., 2023). The observed shift from NFC to FC pool gas production with increased TE inclusion may suggest a redistribution of fermentative activity, as discussed previously. However, the underlying mechanism remains unclear and warrants further investigation, potentially involving carbohydrate fraction analysis or microbial profiling.

The post-fermentation ruminal pH was approximately 6.18, on average, among treatments. Others have also reported that ruminal pH was not affected by supplementation of a CT extract (Norris et al., 2020; Dias Batista et al., 2021), or by a TE containing both HT and CT (Aguerre et al., 2016; Aboagye et al., 2018). Furthermore, the similarities in ivNDFD and total VFA production among inclusion rates are consistent with the findings on ruminal pH. It has been generally recognized that the formation of tannin-nutrient complexes when cattle are supplemented with tannins may lead to reduced nutrient digestibility (Naumann et al., 2017). While reduced nutrient digestibility may contribute to reductions in CH_4_ production, it is not desirable given the potential negative impacts on animal performance. Our results are consistent with those reported by de Jong et al. (2025), in which inclusion of blended CT and HT extract at 0.3% of DM reduced the fermentation rate of forages without reducing digestibility.

Although no differences were observed in total VFA concentrations, a linear decrease in propionate concentration led to a linear increase in the acetate:propionate ratio with increasing TE inclusion rate. When Pellikaan et al. (2011) incubated lucerne hay with different sources of TE in vitro, they found a greater acetate:propionate ratio when incubated with HT compared to CT and the control. In contrast, Beauchemin et al. (2007) reported a linear decrease in the acetate:propionate ratio in ruminal fluid from growing beef cattle supplemented with up to 2% of dietary DM of a CT extract. Typically, a reduction in methanogenesis is accompanied by a decrease in the acetate:propionate ratio, as propionate serves as a competitive hydrogen sink (Moss et al., 2000). In our study, the lowest total CH_4_ production was associated with an inclusion rate of 0.18% of DM, which corresponded to relatively higher ruminal propionate and a lower acetate:propionate ratio, supporting the potential role of the hydrogen sink mechanism. Ultimately, this relationship suggests a possible shift in fermentative pathways, which warrants further investigation. A reduction in CH_4_ production, combined with an increase in ruminal propionate concentration, is an ideal scenario for enhancing energy utilization and potentially increasing the flow of microbial protein from the rumen due to increased energy available for microbial growth (Baker, 1997; Tavendale et al., 2005). Nevertheless, the inconsistencies regarding the effects of TE on ruminal VFA concentration and associated CH_4_ production are likely related to differences in tannin types, sources, and inclusion levels, as well as the fermentation substrate used. In addition, the lack of consistency in the quantification and reporting of TE characteristics limits the comparison of results among studies. Reporting TE inclusion rates and results on a TCT and PPP basis is essential for interstudy comparisons and optimization of TE supplementation for beef cattle.

A possible limitation of this study was the inability to determine the specific proportions of CT and HT in the commercial TE. Future studies should prioritize detailed chemical characterization of TEs, as the relative abundance and bioactivity of each tannin type likely play a central role in modulating ruminal fermentation and CH_4_ production. Additionally, microbial profiling may be crucial for elucidating the underlying mechanisms associated with TE supplementation. As mentioned previously, supplementing beef cattle with TE to mitigate CH_4_ production may have unintended consequences on animal performance, such as reduced feed intake, diet digestibility, and weight gains (Naumann et al., 2017). The IVGP technique serves as a valuable preliminary tool for assessing the potential impacts of feed characteristics and additives on fermentation kinetics and ruminal parameters; however, in vivo studies are essential for a comprehensive understanding of the biological mechanisms occurring within the animal.

## Conclusions

The inclusion of a TE containing both CT and HT affected fermentation kinetics and CH_4_ production, particularly at a low level of inclusion. Although our results are preliminary, supplementation of this TE at a rate of 0.18% of DM appears to offer the most promising potential for reducing CH_4_ production from beef cattle fed a TMR without affecting fiber digestibility or ruminal parameters. Nevertheless, further research is needed to confirm the optimal dose of this commercial TE for mitigating CH_4_ emissions from beef cattle using in vivo studies.

## Abbreviations:

CH_4_: methane
CT: condensed tannins
DM: dry matter
DMI: DM intake
FC: fiber carbohydrate
FOM: fermentable organic matter
GHG: greenhouse gas
HT: hydrolyzable tannins
IVGP: in vitro gas production
ivNDFD: in vitro neutral detergent fiber digestibility
ME: metabolizable energy
NFC: nonfiber carbohydrate
TDN: total digestible nutrients
TE: tannin extract
TMR: total mixed ration
VFA: volatile fatty acids
